# Massive haemothorax from percutaneous nephrolithotomy requiring video-assisted thoracoscopic surgery: A case report

**DOI:** 10.1016/j.ijscr.2023.108251

**Published:** 2023-04-21

**Authors:** Sirawee Ekkasak, Piya Cherntanomwong, Yada Phengsalae, Chinnakhet Ketsuwan

**Affiliations:** Department of Surgery, Faculty of Medicine Ramathibodi Hospital, Mahidol University, Bangkok, Thailand

**Keywords:** Massive haemothorax, PCNL, VATSD

## Abstract

**Introduction:**

Massive haemothorax can occur following percutaneous nephrolithotomy (PCNL), which is a significant adverse event and a life-threatening condition.

**Presentation of case:**

A 65-year-old male who presented with a full right staghorn stone was treated with PCNL. Two days later, he developed massive haemothorax and conservative management with intercostal drainage failed. The patient successfully underwent video-assisted thoracoscopic surgical decortication (VATSD).

**Discussion:**

PCNL is the mainstay procedure for complex renal stones. Because it is aggressive, it can also have serious complications. Tube thoracostomy drainage is the initial approach for managing haemothorax. However, retained haemothorax still occurs and can cause additional complications. VATSD is frequently applied in the modern era because of its good visualization and reduced morbidity compared with conventional thoracotomy.

**Conclusion:**

VATSD is a safe and effective surgical technique that can be easily applied to manage retained haemothorax as a result of PCNL.

## Highlights

• Massive haemothorax can occur following percutaneous nephrolithotomy.
• Intercostal drainage tube insertion is the first choice that can reduce the risk of retained massive haemothorax.
• Video-assisted thoracoscopic surgical decortication is an effective surgical procedure to manage retained haemothorax.

## 1 Introduction

Pulmonary complication that occurs following percutaneous nephrolithotomy (PCNL) is an adverse event of concern, especially in cases of upper pole of the kidney accession [1]. This procedure is performed near the diaphragm; therefore, it may cause injury to the pleural cavity and lungs. Potential chest complications include hydrothorax, haemothorax (HTX), and reno-pleural fistula and sometimes lead to life-threatening conditions [2], [3]. We report the first case of massive HTX from percutaneous nephrolithotomy that showed no improvement following conservative management and intercostal drainage and required video-assisted thoracoscopic surgical decortication (VATSD) for management. This interesting clinical presentation and rationale for management are discussed below. We ensure that the work has been reported in line with the SCARE 2020 criteria [4].

## 2 Presentation of case

**Fig. 1:**
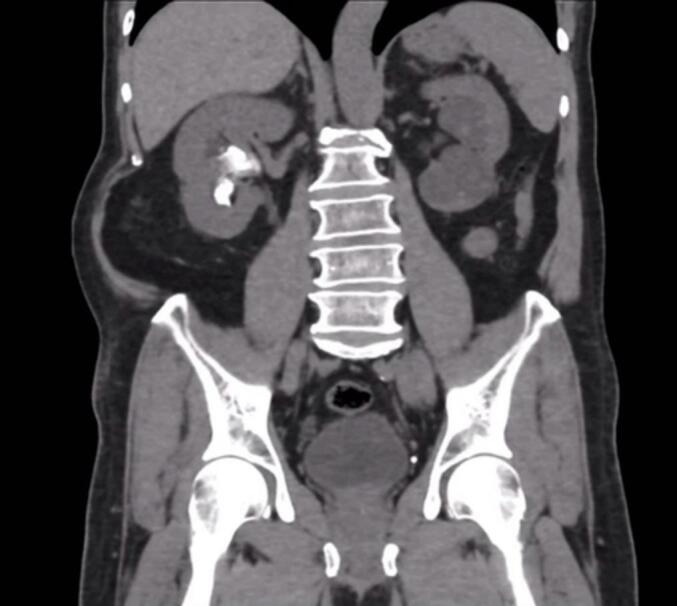
Preoperative CT scan showing a right staghorn stone.

A 65-year-old male presented with a large kidney stone that was detected during a routine medical examination. A computed tomography scan revealed a full right staghorn stone calculus that was 2.4 × 2.2 × 6.5 cm^3^ in size with a density of 680 Hounsfield units (Fig. 1). PCNL was performed using a standard prone approach. The accession was conducted through the upper-pole calyx with the supra-12th rib punctured under fluoroscopic guidance. The stones were fragmented using ultrasonic lithotripsy through a 28F rigid nephroscope. At the end of the procedure, a retrograde 6Fr double-J stent with a nephrostomy tube was placed. Total operative time was 120 min and estimated blood loss (EBL) was 100 mL.

**Fig. 2:**
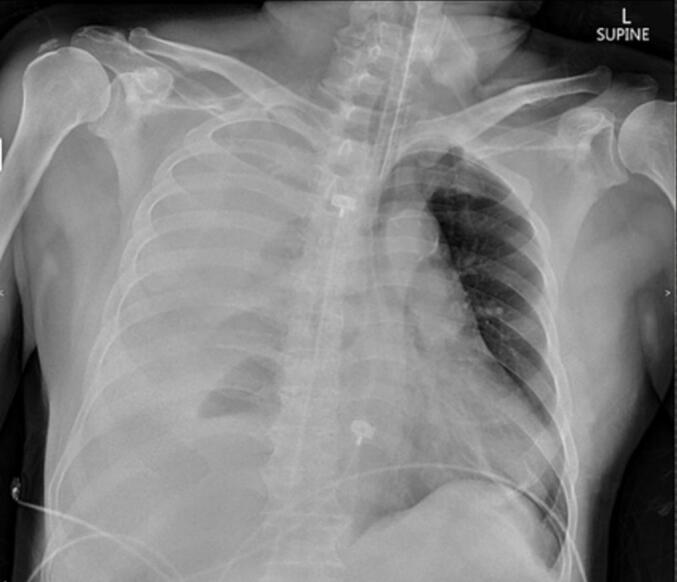
Chest X-ray showing a large amount of right haemothorax.

On postoperative day (POD) 2, the patient developed shortness of breath immediately after withdrawal of the nephrostomy tube. Tachypnea with severe respiratory distress was noted with deteriorating breath sounds on the right side. Chest radiography (CXR) confirmed right pleural effusion (Fig. 2). The patient was intubated and sent to the intensive care unit. A right intercostal drainage (ICD) tube was inserted and a gush of blood approximately 1000 mL streaming through the tube was seen. After adequate fluid resuscitation with blood transfusion, a patient's hemodynamic parameters improved. A renal angiogram was performed, which illustrated a bi-lobed pseudoaneurysm, located in the right renal upper polar artery. Selective embolization of the pseudoaneurysm was accomplished with a microcatheter and two Concerto™ coils were deployed (16 mm × 50 cm and 6 mm × 30 cm).

**Fig. 3:**
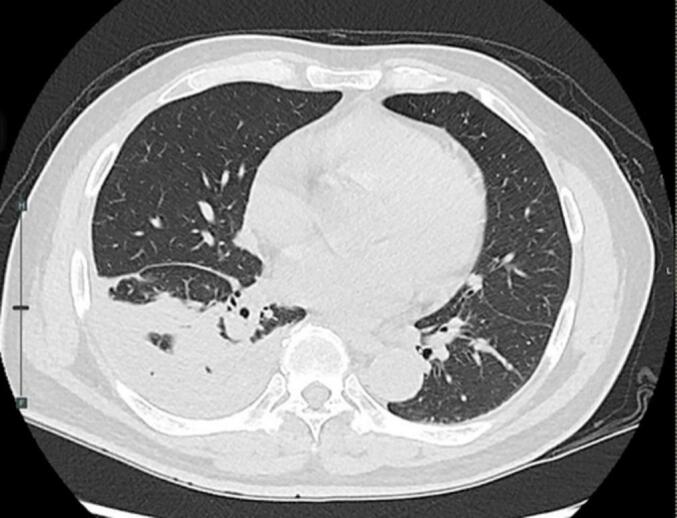
CT scan showing retained haemothorax and passive atelectasis of the lung parenchyma.

Nevertheless, on POD 6, a chest ultrasonography and CXR still illustrated a residual and loculated HTX and then a 16-Fr percutaneous drainage tube was placed under ultrasound guide. A small volume of old blood was drained without an improvement in lung expansion. On POD 10, the patient developed low-grade fever and chest computed tomography scan demonstrated a large amount of retained HTX with atelectasis of the right lung (Fig. 3). Therefore, a decision was made to perform VATSD.

## 3 Surgical technique

**Fig. 4:**
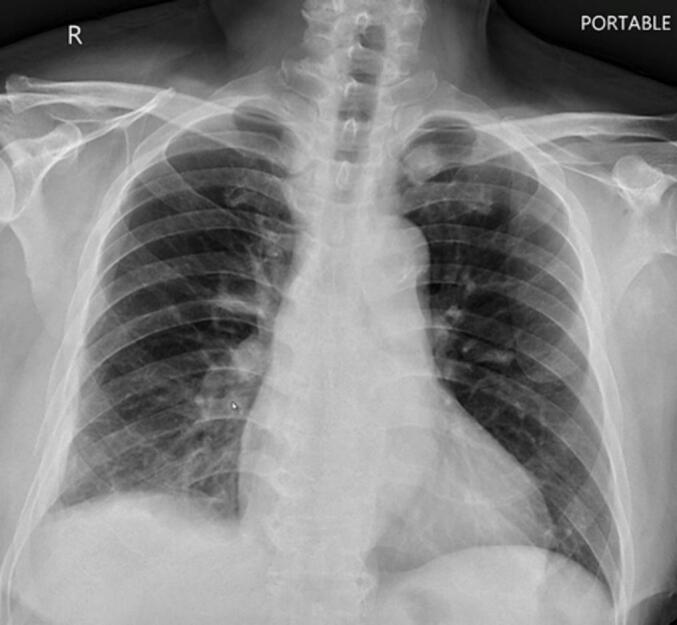
Patient's chest X-ray before discharge.

Under general anaesthesia using one-lung ventilation, the patient was lying in the left lateral decubitus position. The thoracoscopic approach was performed using the two-port technique. The working port was created to be 6 cm in length anterolateral thoracotomy incision into the pleural cavity through the 6th intercostal space. Then, the 1-cm incision for the camera port was made at the 8th intercostal space in the mid-axillary line and was used mainly for introducing a 10-mm 30° thoracoscope. The first step was pleural cavity exploration, which showed a large amount of old clotted blood with largely passive atelectasis of the right lower lobe and the right middle lobe. The blood clot was totally removed using sponge forceps and a curved suction apparatus. After adequate exposure of the lungs, we found no active bleeding point or organ injury. Decortication of the affected pulmonary parenchyma was meticulously performed using an electrocautery device combined with blunt dissection by small peanut-shaped dissecting sponges. Two-lung ventilation was conducted, which revealed no air leaks. Two chest tubes were placed in the thoracic cavity. The total surgery time was 200 min and EBL was 100 mL. The patient was extubated in the operating room and brought to the recovery room. Each chest tube was removed on POD 5 and 7. CXR showed no signs of atelectasis or residual HTX (Fig. 4). The patient was discharged with good functional recovery on POD 10.

## 4 Discussion

Nephrolithiasis affects around 5 % to 15 % of people around the world. PCNL is recognized as the mainstay procedure for the removal of large, multiple and complex renal stones [5], [6]. Although this technique affords high success rates for stone clearance, it also has various complications because of its aggressiveness. The more common perioperative complications of PCNL were extravasation, bleeding requiring a blood transfusion, and fever; major complications, such as septicaemia and colonic or pleural injury that required intervention, were very rare [3], [7]. Various modifications to this technique, including miniature endoscopy or in combination with flexible ureteroscopy, have been developed to decrease adverse events with comparable stone-free rates [5], [8], [9].

Currently, practice management guidelines for the management of HTX recommend ICD insertion as the first choice, which is a feasible and fast procedure [10] that can reduce the risk of retained HTX. However, retained HTX can still occur in 5–30 % of cases [11]. Although these residual HTX may be spontaneously resolved, retained clotted or loculated HTX usually contributes to passive atelectasis of adjacent lung parenchyma and also increases the risk of empyema thoracis and other complications. In the initial phase, large amounts of HTX can compress normal lung tissue, creating ventilation–perfusion mismatch and causing acute respiratory failure. In addition, massive blood loss may affect the patient's hemodynamic and organ perfusion. When retained HTX is contaminated by bacteria, empyema may occur. The incidence of empyema following traumatic HTX is up to 10 % of cases, and retained HTX is one of the significant risk factors, with an incidence of 33 % compared with 2 % among those without retained HTX [12]. In the later phase, fibrothorax may develop. Fibrothorax was proved to influence the pulmonary function test. Overall lung function is similar to that of the restrictive lung pattern, with a significantly reduced vital capacity [13].

The appropriate management for retained HTX is widely discussed, starting from a less invasive procedure, such as placement of additional tube thoracostomy or pigtail catheter, to operative intervention. With technological advancements, imaging-guide PCD using a pigtail catheter by an experienced physician has been proved to be effective in draining HTX and various types of effusion and causing less pain compared with tube thoracostomy [14], [15], [16], [17]. Intrapleural streptokinase is an alternative, less invasive procedure for the treatment of retained HTX; nevertheless, its effectiveness is still inferior to that of video-assisted thoracoscopic surgery (VATS) in terms of the rate of additional operative intervention required [11], [18]. In the past, exploratory thoracotomy was commonly used as the gold standard in the management of retained HTX. However, this approach can be associated with an increase in the length of hospital stay and morbidity compared with a less invasive modality such as VATSD, which is often used in the modern era. Moreover, infectious complications have been shown to be higher in thoracotomy [19]. Since VATS was developed in the 1990s, it has been widely used by thoracic and trauma surgeons due to its better visualization, smaller incision and lower morbidity. Although there are studies that support the position that VATS evacuation of retained HTX and decortication is as effective as open thoracotomy, VATS should be performed only on hemodynamically stable patients [20].

## 5 Conclusion

The refinement of surgical techniques and prompt management of HTX represent a key opportunity to reduce morbidity. This was the first presentation of a massive HTX case that resulted from the PCNL and was successfully managed by VATSD.

## Consent

Written informed consent was obtained from the patient for the publication of this case report and its accompanying images. A copy of the written consent form is available for review by the Editor-in-Chief of this journal on request.

## Ethical approval

The authors' institute provided ethical approval for this case study.

## Funding

We declare that we did not require any funding.

## Author contribution

Sirawee Ekkasak: Conceptualization, data curation, writing of original draft, reviewing and editing

Piya Cherntanomwong: Supervision and reviewing

Yada Phengsalae: Supervision

Chinnakhet Ketsuwan: Writing, reviewing and editing

## Guarantor

Chinnakhet Ketsuwan.

## Research registration number

COA. MURA2023/57.

## Declaration of competing interest

The authors declare that they have no conflicts of interest.
